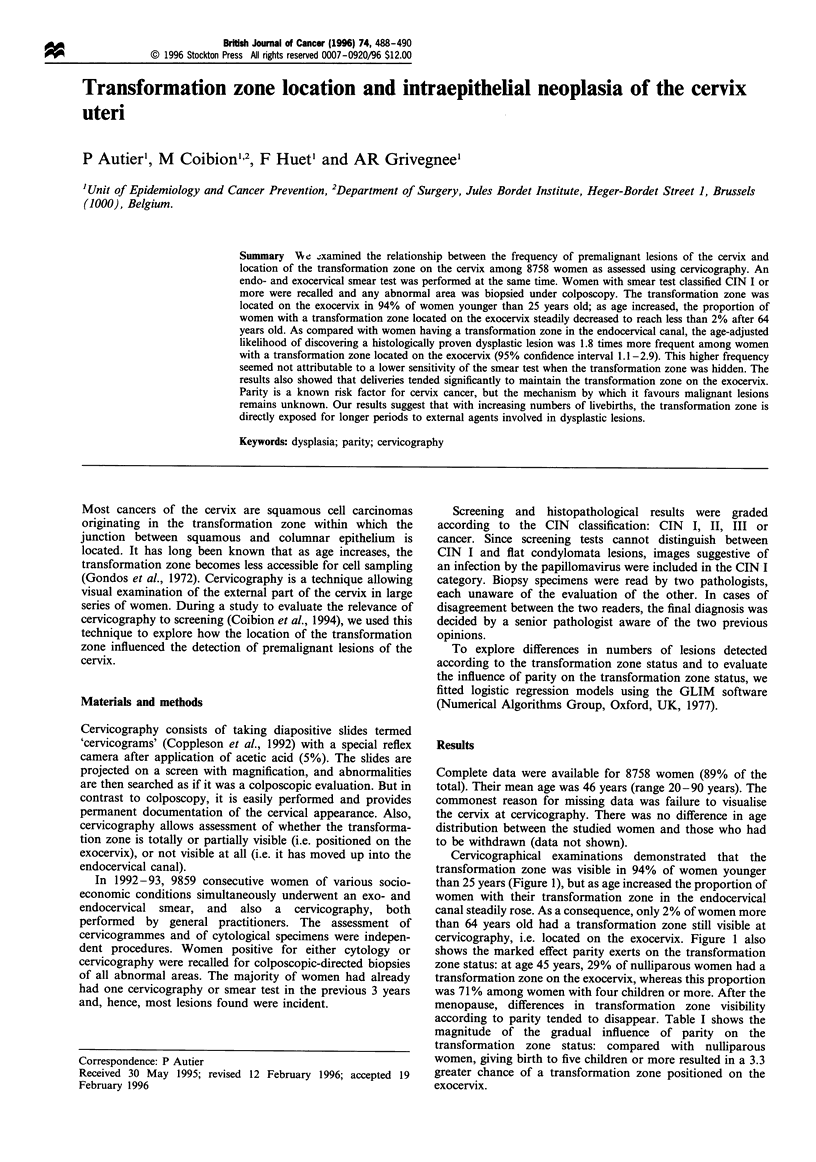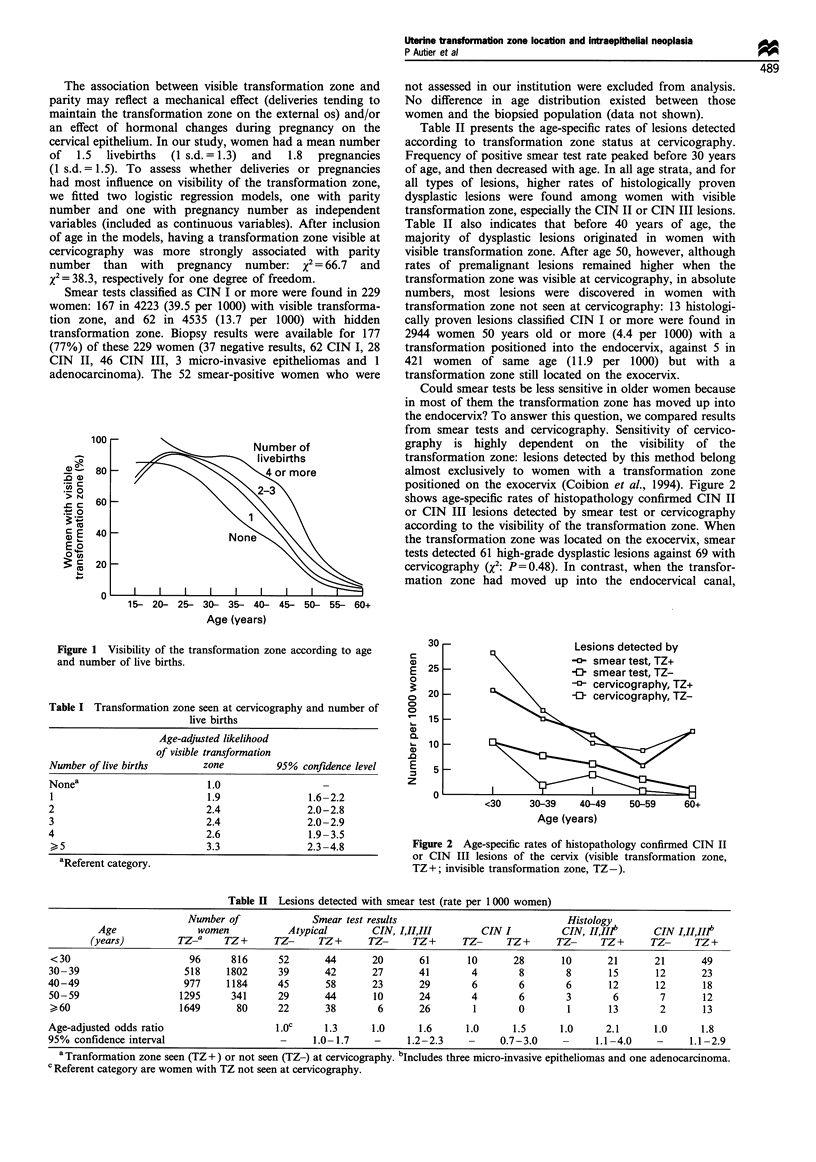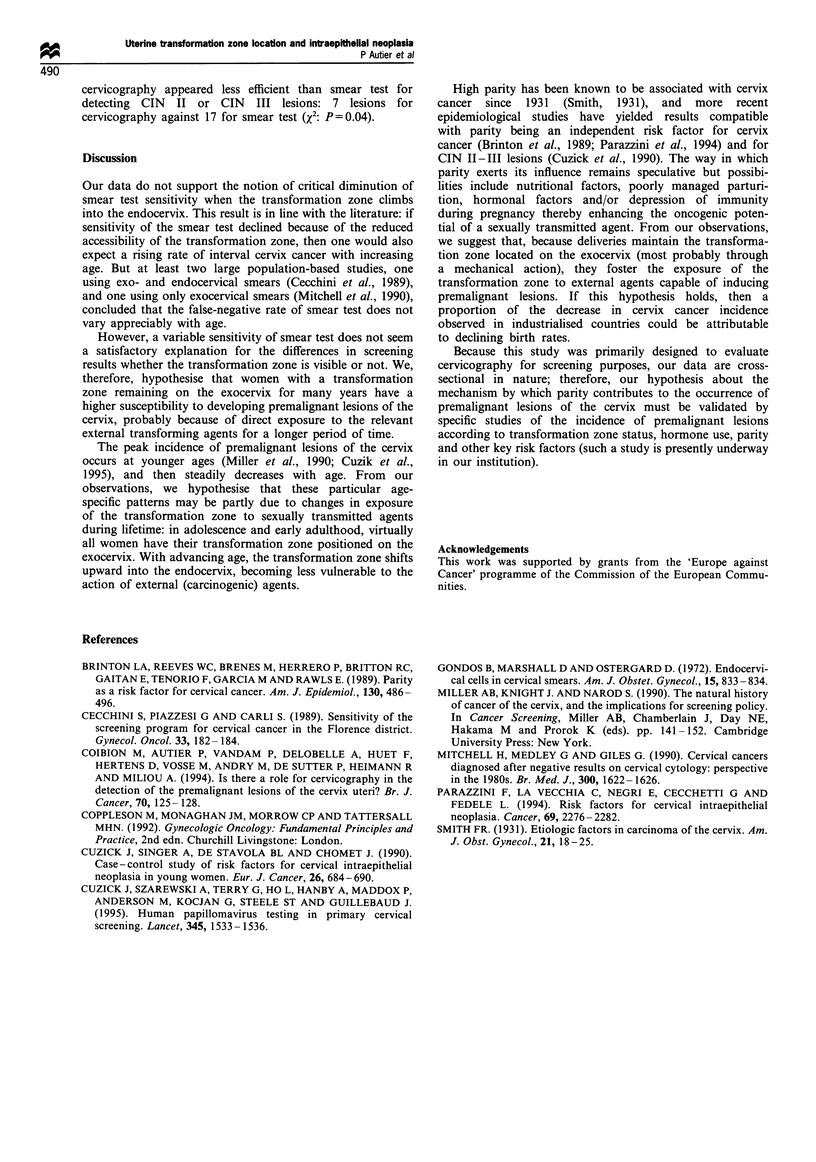# Transformation zone location and intraepithelial neoplasia of the cervix uteri.

**DOI:** 10.1038/bjc.1996.388

**Published:** 1996-08

**Authors:** P. Autier, M. Coibion, F. Huet, A. R. Grivegnee

**Affiliations:** Unit of Epidemiology and Cancer Prevention, Jules Bordet Institute, Brussels, Belgium.

## Abstract

We examined the relationship between the frequency of premalignant lesions of the cervix and location of the transformation zone on the cervix among 8758 women as assessed using cervicography. An endo- and exocervical smear test was performed at the same time. Women with smear test classified CIN I or more were recalled and any abnormal area was biopsied under colposcopy. The transformation zone was located on the exocervix in 94% of women younger than 25 years old; as age increased, the proportion of women with a transformation zone located on the exocervix steadily decreased to reach less than 2% after 64 years old. As compared with women having a transformation zone in the endocervical canal, the age-adjusted likelihood of discovering a histologically proven dysplastic lesion was 1.8 times more frequent among women with a transformation zone located on the exocervix (95% confidence interval 1.1-2.9). This higher frequency seemed not attributable to a lower sensitivity of the smear test when the transformation zone was hidden. The results also showed that deliveries tended significantly to maintain the transformation zone on the exocervix. Parity is a known risk factor for cervix cancer, but the mechanism by which it favours malignant lesions remain unknown. Our results suggest that with increasing numbers of livebirths, the transformation zone is directly exposed for longer periods to external agents involved in dysplastic lesions.


British Journal of Cancer (1996) 74, 488-490
© 1996 Stockton Press All rights reserved 0007-0920/96 $12.00

### Transformation zone location and intraepithelial neoplasia of the cervix uteri

P Autier[1], M Coibion[1,2], F Huet[1] and AR Grivegnee[1]

[1]*Unit of Epidemiology and Cancer Prevention,* [2]*Department of Surgery, Jules Bordet Institute, Heger-Bordet Street 1, Brussels (1000), Belgium.*

**Summary** We examined the relationship between the frequency of premalignant lesions of the cervix and location of the transformation zone on the cervix among 8758 women as assessed using cervicography. An endo- and exocervical smear test was performed at the same time. Women with smear test classified CIN I or more were recalled and any abnormal area was biopsied under colposcopy. The transformation zone was located on the exocervix in 94% of women younger than 25 years old; as age increased, the proportion of women with a transformation zone located on the exocervix steadily decreased to reach less than 2% after 64 years old. As compared with women having a transformation zone in the endocervical canal, the age-adjusted likelihood of discovering a histologically proven dysplastic lesion was 1.8 times more frequent among women with a transformation zone located on the exocervix (95% confidence interval 1.1-2.9). This higher frequency seemed not attributable to a lower sensitivity of the smear test when the transformation zone was hidden. The results also showed that deliveries tended significantly to maintain the transformation zone on the exocervix. Parity is a known risk factor for cervix cancer, but the mechanism by which it favours malignant lesions remains unknown. Our results suggest that with increasing numbers of livebirths, the transformation zone is directly exposed for longer periods to external agents involved in dysplastic lesions.

**Keywords:** dysplasia; parity; cervicography

Most cancers of the cervix are squamous cell carcinomas originating in the transformation zone within which the junction between squamous and columnar epithelium is located. It has long been known that as age increases, the transformation zone becomes less accessible for cell sampling (Gondos *et al.*, 1972). Cervicography is a technique allowing visual examination of the external part of the cervix in large series of women. During a study to evaluate the relevance of cervicography to screening (Coibion *et al.*, 1994), we used this technique to explore how the location of the transformation zone influenced the detection of premalignant lesions of the cervix.

### Materials and methods

Cervicography consists of taking diapositive slides termed 'cervicograms' (Coppleson *et al.*, 1992) with a special reflex camera after application of acetic acid (5%). The slides are projected on a screen with magnification, and abnormalities are then searched as if it was a colposcopic evaluation. But in contrast to colposcopy, it is easily performed and provides permanent documentation of the cervical appearance. Also, cervicography allows assessment of whether the transformation zone is totally or partially visible (i.e. positioned on the exocervix), or not visible at all (i.e. it has moved up into the endocervical canal).

In 1992-93, 9859 consecutive women of various socioeconomic conditions simultaneously underwent an exo- and endocervical smear, and also a cervicography, both performed by general practitioners. The assessment of cervicogrammes and of cytological specimens were independent procedures. Women positive for either cytology or cervicography were recalled for colposcopic-directed biopsies of all abnormal areas. The majority of women had already had one cervicography or smear test in the previous 3 years and, hence, most lesions found were incident.

Correspondence: P Autier
Received 30 May 1995; revised 12 February 1996; accepted 19 February 1996

Screening and histopathological results were graded according to the CIN classification: CIN I, II, III or cancer. Since screening tests cannot distinguish between CIN I and flat condylomata lesions, images suggestive of an infection by the papillomavirus were included in the CIN I category. Biopsy specimens were read by two pathologists, each unaware of the evaluation of the other. In cases of disagreement between the two readers, the final diagnosis was decided by a senior pathologist aware of the two previous opinions.

To explore differences in numbers of lesions detected according to the transformation zone status and to evaluate the influence of parity on the transformation zone status, we fitted logistic regression models using the GLIM software (Numerical Algorithms Group, Oxford, UK, 1977).

### Results

Complete data were available for 8758 women (89% of the total). Their mean age was 46 years (range 20-90 years). The commonest reason for missing data was failure to visualise the cervix at cervicography. There was no difference in age distribution between the studied women and those who had to be withdrawn (data not shown).

Cervicographical examinations demonstrated that the transformation zone was visible in 94% of women younger than 25 years (Figure 1), but as age increased the proportion of women with their transformation zone in the endocervical canal steadily rose. As a consequence, only 2% of women more than 64 years old had a transformation zone still visible at cervicography, i.e. located on the exocervix. Figure 1 also shows the marked effect parity exerts on the transformation zone status: at age 45 years, 29% of nulliparous women had a transformation zone on the exocervix, whereas this proportion was 71% among women with four children or more. After the menopause, differences in transformation zone visibility according to parity tended to disappear. Table I shows the magnitude of the gradual influence of parity on the transformation zone status: compared with nulliparous women, giving birth to five children or more resulted in a 3.3 greater chance of a transformation zone positioned on the exocervix.

The association between visible transformation zone and parity may reflect a mechanical effect (deliveries tending to maintain the transformation zone on the external os) and/or an effect of hormonal changes during pregnancy on the cervical epithelium. In our study, women had a mean number of 1.5 livebirths (1 s.d. = 1.3) and 1.8 pregnancies (1 s.d. = 1.5). To assess whether deliveries or pregnancies had most influence on visibility of the transformation zone, we fitted two logistic regression models, one with parity number and one with pregnancy number as independent variables (included as continuous variables). After inclusion of age in the models, having a transformation zone visible at cervicography was more strongly associated with parity number than with pregnancy number: $\chi^2 = 66.7$ and $\chi^2 = 38.3$, respectively for one degree of freedom.

Smear tests classified as CIN I or more were found in 229 women: 167 in 4223 (39.5 per 1000) with visible transformation zone, and 62 in 4535 (13.7 per 1000) with hidden transformation zone. Biopsy results were available for 177 (77%) of these 229 women (37 negative results, 62 CIN I, 28 CIN II, 46 CIN III, 3 micro-invasive epitheliomas and 1 adenocarcinoma). The 52 smear-positive women who were not assessed in our institution were excluded from analysis. No difference in age distribution existed between those women and the biopsied population (data not shown).

Table II presents the age-specific rates of lesions detected according to transformation zone status at cervicography. Frequency of positive smear test rate peaked before 30 years of age, and then decreased with age. In all age strata, and for all types of lesions, higher rates of histologically proven dysplastic lesions were found among women with visible transformation zone, especially the CIN II or CIN III lesions. Table II also indicates that before 40 years of age, the majority of dysplastic lesions originated in women with visible transformation zone. After age 50, however, although rates of premalignant lesions remained higher when the transformation zone was visible at cervicography, in absolute numbers, most lesions were discovered in women with transformation zone not seen at cervicography: 13 histologically proven lesions classified CIN I or more were found in 2944 women 50 years old or more (4.4 per 1000) with a transformation positioned into the endocervix, against 5 in 421 women of same age (11.9 per 1000) but with a transformation zone still located on the exocervix.

Could smear tests be less sensitive in older women because in most of them the transformation zone has moved up into the endocervix? To answer this question, we compared results from smear tests and cervicography. Sensitivity of cervicography is highly dependent on the visibility of the transformation zone: lesions detected by this method belong almost exclusively to women with a transformation zone positioned on the exocervix (Coibion *et al.*, 1994). Figure 2 shows age-specific rates of histopathology confirmed CIN II or CIN III lesions detected by smear test or cervicography according to the visibility of the transformation zone. When the transformation zone was located on the exocervix, smear tests detected 61 high-grade dysplastic lesions against 69 with cervicography ($\chi^2$: $P = 0.48$). In contrast, when the transformation zone had moved up into the endocervical canal,

**Figure 1** Visibility of the transformation zone according to age and number of live births.

**Table I** Transformation zone seen at cervicography and number of live births

| Number of live births | Age-adjusted likelihood of visible transformation zone | 95% confidence level |
|---|---|---|
| None[a] | 1.0 | – |
| 1 | 1.9 | 1.6–2.2 |
| 2 | 2.4 | 2.0–2.8 |
| 3 | 2.4 | 2.0–2.9 |
| 4 | 2.6 | 1.9–3.5 |
| ⩾5 | 3.3 | 2.3–4.8 |

[a]Referent category.

**Figure 2** Age-specific rates of histopathology confirmed CIN II or CIN III lesions of the cervix (visible transformation zone, TZ+; invisible transformation zone, TZ–).

**Table II** Lesions detected with smear test (rate per 1 000 women)

| Age (years) | Number of women TZ–[a] | TZ+ | Smear test results: Atypical TZ– | TZ+ | CIN, I,II,III TZ– | TZ+ | Histology: CIN I TZ– | TZ+ | CIN, II,III[b] TZ– | TZ+ | CIN I,II,III[b] TZ– | TZ+ |
|---|---|---|---|---|---|---|---|---|---|---|---|---|
| <30 | 96 | 816 | 52 | 44 | 20 | 61 | 10 | 28 | 10 | 21 | 21 | 49 |
| 30–39 | 518 | 1802 | 39 | 42 | 27 | 41 | 4 | 8 | 8 | 15 | 12 | 23 |
| 40–49 | 977 | 1184 | 45 | 58 | 23 | 29 | 6 | 6 | 6 | 12 | 12 | 18 |
| 50–59 | 1295 | 341 | 29 | 44 | 10 | 24 | 4 | 6 | 3 | 6 | 7 | 12 |
| ⩾60 | 1649 | 80 | 22 | 38 | 6 | 26 | 1 | 0 | 1 | 13 | 2 | 13 |
| Age-adjusted odds ratio | | | 1.0[c] | 1.3 | 1.0 | 1.6 | 1.0 | 1.5 | 1.0 | 2.1 | 1.0 | 1.8 |
| 95% confidence interval | | | – | 1.0–1.7 | – | 1.2–2.3 | – | 0.7–3.0 | – | 1.1–4.0 | – | 1.1–2.9 |

[a]Tranformation zone seen (TZ+) or not seen (TZ–) at cervicography. [b]Includes three micro-invasive epitheliomas and one adenocarcinoma. [c]Referent category are women with TZ not seen at cervicography.

cervicography appeared less efficient than smear test for detecting CIN II or CIN III lesions: 7 lesions for cervicography against 17 for smear test ($\chi^2$: $P=0.04$).

### Discussion

Our data do not support the notion of critical diminution of smear test sensitivity when the transformation zone climbs into the endocervix. This result is in line with the literature: if sensitivity of the smear test declined because of the reduced accessibility of the transformation zone, then one would also expect a rising rate of interval cervix cancer with increasing age. But at least two large population-based studies, one using exo- and endocervical smears (Cecchini *et al.*, 1989), and one using only exocervical smears (Mitchell *et al.*, 1990), concluded that the false-negative rate of smear test does not vary appreciably with age.

However, a variable sensitivity of smear test does not seem a satisfactory explanation for the differences in screening results whether the transformation zone is visible or not. We, therefore, hypothesise that women with a transformation zone remaining on the exocervix for many years have a higher susceptibility to developing premalignant lesions of the cervix, probably because of direct exposure to the relevant external transforming agents for a longer period of time.

The peak incidence of premalignant lesions of the cervix occurs at younger ages (Miller *et al.*, 1990; Cuzik *et al.*, 1995), and then steadily decreases with age. From our observations, we hypothesise that these particular age-specific patterns may be partly due to changes in exposure of the transformation zone to sexually transmitted agents during lifetime: in adolescence and early adulthood, virtually all women have their transformation zone positioned on the exocervix. With advancing age, the transformation zone shifts upward into the endocervix, becoming less vulnerable to the action of external (carcinogenic) agents.

High parity has been known to be associated with cervix cancer since 1931 (Smith, 1931), and more recent epidemiological studies have yielded results compatible with parity being an independent risk factor for cervix cancer (Brinton *et al.*, 1989; Parazzini *et al.*, 1994) and for CIN II–III lesions (Cuzick *et al.*, 1990). The way in which parity exerts its influence remains speculative but possibilities include nutritional factors, poorly managed parturition, hormonal factors and/or depression of immunity during pregnancy thereby enhancing the oncogenic potential of a sexually transmitted agent. From our observations, we suggest that, because deliveries maintain the transformation zone located on the exocervix (most probably through a mechanical action), they foster the exposure of the transformation zone to external agents capable of inducing premalignant lesions. If this hypothesis holds, then a proportion of the decrease in cervix cancer incidence observed in industrialised countries could be attributable to declining birth rates.

Because this study was primarily designed to evaluate cervicography for screening purposes, our data are cross-sectional in nature; therefore, our hypothesis about the mechanism by which parity contributes to the occurrence of premalignant lesions of the cervix must be validated by specific studies of the incidence of premalignant lesions according to transformation zone status, hormone use, parity and other key risk factors (such a study is presently underway in our institution).

**Acknowledgements**

This work was supported by grants from the 'Europe against Cancer' programme of the Commission of the European Communities.

### References

BRINTON LA, REEVES WC, BRENES M, HERRERO P, BRITTON RC, GAITAN E, TENORIO F, GARCIA M AND RAWLS E. (1989). Parity as a risk factor for cervical cancer. *Am. J. Epidemiol.*, **130**, 486–496.

CECCHINI S, PIAZZESI G AND CARLI S. (1989). Sensitivity of the screening program for cervical cancer in the Florence district. *Gynecol. Oncol.* **33**, 182–184.

COIBION M, AUTIER P, VANDAM P, DELOBELLE A, HUET F, HERTENS D, VOSSE M, ANDRY M, DE SUTTER P, HEIMANN R AND MILIOU A. (1994). Is there a role for cervicography in the detection of the premalignant lesions of the cervix uteri? *Br. J. Cancer*, **70**, 125–128.

COPPLESON M, MONAGHAN JM, MORROW CP AND TATTERSALL MHN. (1992). *Gynecologic Oncology: Fundamental Principles and Practice*, 2nd edn. Churchill Livingstone: London.

CUZICK J, SINGER A, DE STAVOLA BL AND CHOMET J. (1990). Case–control study of risk factors for cervical intraepithelial neoplasia in young women. *Eur. J. Cancer*, **26**, 684–690.

CUZICK J, SZAREWSKI A, TERRY G, HO L, HANBY A, MADDOX P, ANDERSON M, KOCJAN G, STEELE ST AND GUILLEBAUD J. (1995). Human papillomavirus testing in primary cervical screening. *Lancet*, **345**, 1533–1536.

GONDOS B, MARSHALL D AND OSTERGARD D. (1972). Endocervical cells in cervical smears. *Am. J. Obstet. Gynecol.*, **15**, 833–834.

MILLER AB, KNIGHT J. AND NAROD S. (1990). The natural history of cancer of the cervix, and the implications for screening policy. In *Cancer Screening*, Miller AB, Chamberlain J, Day NE, Hakama M and Prorok K (eds). pp. 141–152. Cambridge University Press: New York.

MITCHELL H, MEDLEY G AND GILES G. (1990). Cervical cancers diagnosed after negative results on cervical cytology: perspective in the 1980s. *Br. Med. J.*, **300**, 1622–1626.

PARAZZINI F, LA VECCHIA C, NEGRI E, CECCHETTI G AND FEDELE L. (1994). Risk factors for cervical intraepithelial neoplasia. *Cancer*, **69**, 2276–2282.

SMITH FR. (1931). Etiologic factors in carcinoma of the cervix. *Am. J. Obst. Gynecol.*, **21**, 18–25.